# Expanding general practice with interprofessional teams: a mixed-methods patient perspective study

**DOI:** 10.1186/s12913-023-10322-z

**Published:** 2023-11-30

**Authors:** Birgit Abelsen, Kine Pedersen, Hanna Isabel Løyland, Emilie Aandahl

**Affiliations:** 1 Department of Community Medicine, National Centre for Rural Medicine, UiT The Arctic University of Norway, Postbox 6050 Langnes, 9037 Tromsø, Norway; 2 Oslo Economics, Klingenberggata 7, Oslo, 0161 Norway

**Keywords:** Interprofessional primary healthcare team, General practice, General practitioner, Registered nurse, Teamwork, Continuity, Patient perspective, Patient-reported experience

## Abstract

**Background:**

Across healthcare systems, current health policies promote interprofessional teamwork. Compared to single-profession general practitioner care, interprofessional primary healthcare teams are expected to possess added capacity to care for an increasingly complex patient population. This study aims to explore patients’ experiences when their usual primary healthcare encounter with general practice shifts from single-profession general practitioner care to interprofessional team-based care.

**Methods:**

Qualitative and quantitative data were collected through interviews and a survey among Norwegian patients. The interviews included ten patients (five women and five men) aged between 28 and 89, and four next of kin (all women). The qualitative analysis was carried out using thematic analysis and a continuity framework. The survey included 287 respondents, comprising 58 per cent female and 42 per cent male participants, aged 18 years and above. The respondents exhibited multiple diagnoses and often a lengthy history of illness. All participants experienced the transition to interprofessional teamwork at their general practitioner surgery as part of a primary healthcare team pilot.

**Results:**

The interviewees described team-based care as more fitting and better coordinated, including more time and more learning than with single-profession general practitioner care. Most survey respondents experienced improvements in understanding and mastering their health problems.

Multi-morbid elderly interviewees and interviewees with mental illness shared experiences of improved information continuity. They found that important concerns they had raised with the nurse were known to the general practitioner and vice versa.

None of the interviewees expressed dissatisfaction with the inclusion of a nurse in their general practitioner relationship. Several interviewees noted improved access to care. The nurse was seen as a strengthening link to the general practitioner. The survey respondents expressed strong agreement with being followed up by a nurse. The interviewees trusted that it was their general practitioner who controlled what happened to them in the general practitioner surgery.

**Conclusion:**

From the patients’ perspective, interprofessional teamwork in general practice can strengthen management, informational, and relational continuity. However, a prerequisite seems to be a clear general practitioner presence in the team.

**Supplementary Information:**

The online version contains supplementary material available at 10.1186/s12913-023-10322-z.

## Background

Interprofessional primary healthcare teams (PHTs), consisting of general practitioners (GPs) and other health professions, are increasingly advocated as an alternative to single-profession GP practice. The teams are expected to have greater capacity to focus on preventive care, chronic disease management, and a more suitable division of labour among various relevant professions [1]. A key feature in European health systems has been the GP as the entry point and gatekeeper. However, a trend can be observed of PHT practices developing from GP practices [2]. Nurses are most often the expanding ingredient, although there are large variations in the composition of PHTs throughout Europe [3, 4]. Groenewegen argues that “nurses are the grease in the primary care innovation machinery” [5], and evidence supports the notion that increased numbers and new roles for nurses lead to improved healthcare outcomes in primary care [4, 6–9].

Limited research has been conducted investigating patients’ perceptions of the transition from GP care to interprofessional team-based care and their valuing of the results [10–13]. Without paying attention to patients’ perceptions of PHTs, changes like this risk being disproportionately orientated towards service provider assessments and political objectives and disconnected from the realities of the patients’ everyday lives.

PHT reforms expanding the patient-GP relationship to other health professionals may be disruptive to continuity of care [14, 15]. Continuity of care has been an essential quality feature of primary healthcare [16] and becomes increasingly important for patients as they age, develop multiple morbidities and complex problems, and/or become socially or psychologically vulnerable [17]. Continuity is not an end but a means to an end of good-quality patient care. It is the degree to which a series of discrete healthcare events are experienced as coherent and connected, and consistent with the patient’s medical needs and personal context [18]. Continuity can be seen from different perspectives, e.g. those of patients, providers, and organizations. In this study, we aim to see continuity from the patient’s perspective. Our attention is on how individual patients experience changes in the integration of services and coordination as their primary healthcare service shifts from single-profession GP care to team-based care provided by a GP and a nurse.

Continuity of care can be decomposed into three types: management, informational, and relational [18, 19]. Management continuity is relevant whenever a patient is receiving care from more than one provider. It concerns the processes involved in coordinating, integrating, and personalizing care to ensure a consistent course of treatment over time and across different providers [17]. Informational continuity links care from one provider to another and from one healthcare event to another. Information can be disease or person focused. Documented information tends to focus on the medical condition but knowledge about the patient’s preferences, values, and context is equally important for bridging separate care events and ensuring that services are responsive to patient needs. Knowledge about the patient’s preferences, values, and context is often tacit and usually accumulated in the memory of providers who interact with the patient [18]. Relational continuity refers to a provider-patient relationship over time and across different health events. In the primary healthcare research literature, relational continuity is often conceived as a relationship between a patient and a GP. The patient-GP relationship is part of a larger context, and design and results depend on national conditions and the structure of the health service [19].

Murphy and Salisbury [20] claim that in the UK, relational continuity has been consistently decreasing over the last decade, because of falling numbers of GPs, rising workload, increasing complexity, and policies that prioritize access over continuity. Access to care is a highly valued goal in health policy, and PHTs are promoted as an aim to improve patients’ access to primary healthcare. Access to care is sometimes seen in contrast to continuity of care in the sense that it might be difficult due to time constraints to achieve both in single-profession general practice settings [19].

The present study utilizes the framework presented above concerning continuity of care and aim to explore how patients’ experience changes in continuity when GP care expands to interprofessional team-based care which in our example involves care provided by GP and nurse. The aim is also to understand patients’ preferences for team-based care in general practice.

## Methods

### Study context

Since 2001, Norway has had a patient list system in general practice. This system gives every 5.4 mill. Norwegian citizens the right to be listed with a regular GP, and only a minimal number of people opt out [21]. The primary objective with the list system is to secure access and continuity of care. Regular GPs provide consultations with their listed patients and coordinate their care within the healthcare system, while also serving as gatekeepers to secondary care and sickness benefits.

Over the years the general population has been very satisfied with the list system [22]. Delalic et al. [21] show that in 2021, 80 per cent of consultations for patients listed with a regular GP were with their regular GP. The percentage was higher for patients living in the most central municipalities and lower for those living in the most rural (range: 71–81 per cent). An earlier study shows that the percentage of consultations with the regular GP is highest among elderly patients and those with a high number of consultations, indicating chronic conditions [23].

Even though the regular GP scheme has been one of the most popular social services in Norway over the past several years, multiple reports have pointed to areas of improvement [24, 25]. There was a need for measures to improve the availability of the service (e.g., reduce the waiting time for a GP appointment), offer a broader range of services at the GP office, improve follow-up of patients with chronic illnesses and patients with large and complex needs, and to establish more coherent and coordinated services.

In 2022, regular GPs had on average 1,040 listed patients [26]. They typically work in relatively small practices alongside other regular GPs (mean number: 5, interquartile range: [3–6]) [27], and it is uncommon for practices to employ professionals other than medical assistants. Thus, interprofessional teamwork in general practice is less developed in Norway than in many other European countries [3].

The Norwegian Directorate of Health initiated a primary healthcare team (PHT) pilot in the period April 2018 – March 2023. The aim of the PHT pilot was to try out team organization based in general practices. The pilot expanded general practices with nurses and a PHT includes regular GPs, nurses, and medical assistants [28].

The PHT pilot provided funding for the nurse resource in the approximate ratio of one nurse per three regular GPs. The pilot included 17 self-recruited general practices, each of which included between three and 17 regular GPs, one to four nurses, and between two and six medical assistants.

The target patient groups for the PHTs were weak demanders and patients with large and complex needs grouped in four main categories: patients with chronic diseases; patients with mental health problems and/or substance abuse problems; frail elderly; and patients with developmental disorders/disabilities.

### Mixed methods

We utilized a mixed-methods approach [29] with an exploratory sequential design where qualitative information was collected from interviews and quantitative information from a survey during different phases of the pilot. The interviews were conducted and analyzed as a first separate study. Integration through building occurred at the study design level as findings from the interview study informed the survey design. The findings also informed the interpretation of the survey data in the second study. Quantitative and qualitative findings from the two separate studies were in the final phase integrated through narratives using a weaving approach. All authors participated in the final phase of the mixed-method analysis, and it is the results from the final phase we report in this article.

### The qualitative study

#### Participants

The qualitative data collection was carried out among a small sample of patients included in the PHT pilot. The patients had experienced a transition from being followed up by their regular GP to receiving follow-up from a PHT. This means that the patients could compare the situation before and after.

The study participants included patients listed with regular GPs in four of the 17 PHTs. The study PHTs were selected by the authors for convenience. The interviewees were chosen by the PHTs. The authors asked for help to recruit patients in the target groups who had experienced services from PHTs over time, i.e. beyond a single consultation, who were able to express themselves orally, and who seemed comfortable with being interviewed about their PHT experiences. In addition, the interviewees had to have a stable relationship with their regular GP and had to be followed up before the PHT was established to be able to make before/after assessments.

#### Data production

The interviews were conducted between December 2019 and March 2020. The patient interviews were performed face to face with one interviewer present in the interviewee’s home or in the GP surgery. They lasted between 23 and 56 min (mean: 38 min). Four patients insisted on including a next of kin to supplement their own information. Two wives were present in two patient interviews, while two interviews with one mother and one daughter were carried out after the patient interviews. These two next-of-kin interviews were conducted by phone and lasted 10 and 13 min, respectively. We saw no reason not to comply with the interviewees' wish to include their next of kin. Especially in the two cases where the patient's wives were present, we understood this as a prerequisite for the interviews to be carried out. The information the next of kins contributed was essentially a repetition of the information the patients gave themselves. The interview guide was developed for this study (see supplementary file). The interviews took the form of a mainly open conversation, guided by a semi-structured schedule with key topic areas relating to patients’ experience of receiving follow-up from the PHT, their experience of accessibility and continuity, and their perceived values of follow-up from the PHT compared to that from their regular GP. Interviews were audio-recorded and transcribed verbatim.

#### Analysis

The interview transcripts were analysed by BA and MG using an initial inductive approach and thematic analysis [30]. The steps included familiarizing with the data, generating initial codes, further categorization of the coded data, searching for themes, reviewing themes, defining as well as naming themes, and the write-up. After the initial coding the analysis was done in dialog with the previously outlined continuity framework with the three core themes of managerial, informational, and relational continuity introducing a deductive element in the analysis. Table 1 provides examples of how we condensed meanings, from units of meanings as quotations via initial themes and subthemes, to the core themes. The analysis was conducted at a semantic level.

**Table 1:** Examples of meaning condensation from meaning units to initial themes, subthemes, and core themes

No	Meaning unit (quotes)	Initial theme	Sub-theme	Core theme
4	*First, [the nurse] has time. I have no doubt that my GP could come up with good advice. She is very, very skilled. But as a GP, she can't sit and talk to me for an hour*	Time to present problems	More consultation time	Management continuity
13	*We talked through everything, tried out medication, and then my GP came in occasionally, and just wanted to talk. I liked that dialogue, talking and exchanging. I found out a lot about myself, my disease picture and what I should do. It was more than 10 min with the GP to put it that way*	Time to decide on treatment		
4	*I have said many times just before we finish, ‘oh it was so good to see you, now I know what to do’. She [the nurse] may have come up with something, or I just understand what to do based on the conversation we have had*	Making progress	More fitted	
1	*I don't think she [the nurse] stands in the way of my contact with the GP, she [the nurse] is more of a link between me and the GP*	Nurse as a link to the GP	Linkage	Relational continuity
2	*I think they have such good cooperation. […] It's not like he [the GP] says that and she [the nurse] says that they fill each other out and are a team without equal*	Good teamwork	Teamwork	
10	*Now I have the same people to relate to. I know them in and out and they know me*	Knowing each other	Continuity	

### The quantitative study

#### Participants

The target population for the survey was patients with at least one recent (last two months) consultation with a PHT nurse. The 17 GP surgeries in the PHT pilot received the patient survey in August 2022 and were requested to distribute the survey to eligible patients until early October. One GP surgery opted not to participate. A total of 614 eligible patients were given a questionnaire and asked to participate in the survey.

#### Data production

The survey was distributed by a nurse or a medical assistant at the GP surgeries to ensure that the patients qualified as respondents. Eligible patients received a paper questionnaire along with an information sheet about the trial. Additionally, a pre-addressed and prepaid envelope was provided for respondents to return the completed questionnaire to the researchers. The patients could also respond digitally to the survey. The survey was distributed by a nurse or a medical assistant at the GP surgeries to ensure that the patients qualified as respondents, since a previous patient survey revealed that some of the patients did not know whether they received care from a PHT nurse [31].

The survey (see supplementary file) was designed based on questions from the survey *Patients' Experiences with the General Practitioner and the General Practitioner's Office in 2021/2022* [32]. We also developed new questions for this study. The survey topics included the type and frequency of contact with, and follow-up from, the GP surgery, the degree of satisfaction with the follow-up from the nurse, the GP, and the GP surgery overall, the importance and perceived consequences of receiving care from a PHT for the patient, as well as preferences for receiving healthcare. Background information about the respondents was also collected.

The respondents answered a maximum of 36 questions, several of which were conditional based on their previous answers. The respondents could report on consultations both with and without the GP present, and they could report on either type or both types of consultation. Likert scale statements or questions were mostly used, followed by a series of six answer statements (e.g. five grading levels of agreement or levels of extent and a not relevant/do not know option).

#### Analysis

The responses from the printed surveys were manually converted into digital format. Some answers were either unclear or filled out in a way that could not be transferred to a digital format. Therefore, the researchers established rules of assessment to remain as neutral as possible in the assessments. For instance, if the respondent had chosen two options instead of one, the researchers selected the option believed to be most neutral (e.g. if both pretty good and very good were selected, the researcher selected pretty good). The responses from the printed and digital surveys were subsequently combined into a single data set. For each statement/question, we tabulated the frequency distribution of responses using STATA statistical software version 17. The results were used as a descriptive statistical measure to indicate percentage distribution of answers. The survey was carried out by KP, HIL and EA.

### Ethics

The Norwegian Centre for Research Data was notified of the interview study (#405955) and the survey (#547568). In addition, the Regional Committee for Medical and Health Research Ethics South-East (REC) was notified of the interview study. The REC indicated that the project did not require approval from them (2019/28436). All participants gave written consent before they participated in an interview or survey.

## Results

### Participant characteristics

The interview study included 14 participants, i.e. ten patients and four next of kin (two wives, one mother, and one daughter). The patients included five men and five women aged between 28 and 89 years. Most of them had multiple diagnoses and a lengthy disease history. Their diseases and other issues are listed in Table 2.

**Table 2:** Characteristics of the interviewees: 10 patients and 4 next of kin

No	Sex	Age	Role	Medical problem(s)
1	Male	52	Patient	Chronic disease
2	Female	70	Patient	Chronic disease
3	Male	59	Patient	Multiple chronic diseases
4	Female	54	Patient	Psychological, huge caring task for next of kin
5	Female	76	Patient	Compound, e.g. wound, immobility
6	Male	87	Patient	Multiple chronic diseases, immobility
7	Female		Next of kin (wife of no. 6)	
8	Female	28	Patient	Psychological
9	Female		Next of kin (mother of no. 8)	
10	Male	32	Patient	Psychological, substance abuse, obesity
11	Female	89	Patient	Chronic disease, psychological, immobility
12	Female		Next of kin (daughter of no. 11)	
13	Male	72	Patient	Multiple chronic diseases, obesity
14	Female		Next of kin (wife of no. 13)	

The survey included 287 respondents (46 per cent response rate), 256 of whom filled out the survey on paper while 31 responded digitally. The response rate varied from 8 to 82 per cent across the different GP surgeries. Among the respondents, 58 per cent were female and 42 per cent were male. The respondents were in age groups from 18 years and above, and less than half of them defined their health as pretty good/very good (46.8 per cent), a lower share than in the general population (67 per cent) [33]. The most frequent medical conditions were issues related to muscles and the skeleton (41 per cent) and cardiovascular diseases (54 per cent). In total, about 72 per cent of the patients selected more than two of the medical condition options (the options “currently have no long-term health-related problems/conditions” and “do not want to answer” are excluded from this calculation). See Tables 3 and 4 for more details.

**Table 3:** Characteristics of the survey respondents, *n* = 287

Variable	Value	Percent
**Age (*n* = 283)**	18–39 years	9.9
	40–59 years	21.2
	60–79 years	51.9
	80 years or older	16.6
	Do not wish to answer	0.4
**Gender (*n* = 283)**	Male	42.0
	Female	58.0
**Education level (*n* = 278)**	Elementary school	20.1
	High school	39.6
	College/university (up to 3 years)	17.6
	College/university (4 years or more)	11.2
	Do not wish to answer	11.5
**Previous long-term medical conditions (*n* = 284)**	Cardiovascular disease	53.5
	Diabetes	36.3
	Asthma or other chronic lung diseases like chronic bronchitis, emphysema, or COPD	27.5
	Mental health issues, including drug problems	23.2
	Cancer	8.8
	Problems with muscle and/or skeleton, including joints or arthritis	41.2
	Currently have other long-term health-related problems/conditions	29.9
	Currently have no long-term health-related problems/conditions	3.9
	Do not wish to answer	2.1
**Self-rated health (*n* = 280)**	Very bad	2.1
	Pretty bad	10.7
	Neither bad nor good	38.2
	Pretty good	38.9
	Very good	7.9
	Do not wish to answer	2.1

**Table 4:** The distribution of answers to statements concerning the importance of PHT for the respondent and preferences for healthcare services, ranging from disagree to agree (percentage distribution)

	**Disagree**	**Partly disagree**	**Neither agree** **nor disagree**	**Partly agree**	**Agree**	**Not relevant/do not know**	**n**
The collaboration between GP and nurse in the follow-up on my health works well	1.1	1.1	3.2	9.2	74.1	11.3	282
The GP and nurse work together as a team in my follow-up	0.7	1.1	3.6	7.9	71.4	15.4	280
I do not want to be followed up by a nurse at the GP surgery	74.4	4.0	5.5	2.6	4.8	8.8	273
The nurse has more time for me than my regular GP	10.5	4.7	18.4	18.4	40.8	7.2	277
My regular GP understands my health-related issues better than a nurse	11.3	9.1	34.5	15.6	17.8	11.6	275
It is easier for me to address topics I have questions about with a nurse than with my regular GP	22.3	7.7	31.0	14.6	13.1	11.3	274
The nurse contributes with useful advice that I would not have received from my regular GP	9.8	9.1	29.3	21.4	19.6	10.9	276

### Management continuity

The interviewees were mostly focused on the follow-up that can be labelled under management continuity. The PHT follow-up was characterized by a greater degree of planning in patient treatment, as well as more coordination on the part of the GP surgery. The nurses had provided something new and additional to the GP’s follow-up that the interviewees greatly appreciated.

In the interviews, patients with chronic diseases such as diabetes and COPD talked about the establishment of planned and systematic follow-up and alternating consultation with the GP and nurse. Interviewees in need of lifestyle changes talked about the establishment of adapted systematic processes with frequent contact, mainly with the nurse. Elderly, multi-morbid interviewees talked about a new and frequent contact with the nurse, who often now comes to their home to carry out procedures and examinations for which they previously had to go to the GP surgery.

Interviewees talked about different aspects of increased well-being and ability to self-manage as a result of the PHT follow-up and used expressions like “feeling safe”, “taken care of”, “seen and understood”, “happier”, “more motivated to cope”, and “more competent to cope”.

During the past 12 months, the most common frequency of consultations for the respondents was two to five consultations with the nurse where the GP (partly) participated (35.0 per cent). Similarly, for consultations solely with the nurse without the GP’s involvement, two to five consultations was also the most commonly reported frequency (39.8 per cent). The nurses mostly had conversations with the respondents (49.2 per cent), collected blood samples (44.3 per cent), and gave information and training related to their health issues (43.1 per cent).

#### More consultation time

The vast majority among the interviewees talked about time and the fact that PHT follow-up gave them more consultation time than before. This finding is further supported by the survey results, where most respondents reported that the nurse has sufficient time for them (35.5 per cent to a large extent, 58.1 per cent to a very large extent), and a large proportion agreed that the nurse has more time for them than the GP does (18.4 per cent partly agree and 40.8 per cent agree; see Table 4).

The interviewees experienced that the GP has very limited time to spare them and that they must be sharp and focused on medical issues when consulting the GP. The extra time accompanying PHT follow-up – mainly with a nurse – gave them, first, a better opportunity to present their medical problems, second, a better opportunity to participate in treatment decisions, and third, time to properly carry out treatment. The interviewees mainly talked about the benefit of the nurses giving them more time than they get with the GP, but also in some cases about how satisfying it was when the GP and the nurse set aside consultation time for them together.

One interviewee described how the speedy GP consultations often ended with him having to come back for a new consultation because the GP did not have time to hear him out. The GP consultations were still much like that, but the interviewee found the situation more satisfying because he could follow this up with a subsequent consultation with a nurse who had more time for him where there was “*no rushing in and out*”. A mentally ill interviewee emphasized how important it was that the nurse had assured her that they were in no hurry to find a suitable treatment plan that was right for her. Her mother elaborated on this against the background of her daughter's previous treatment experience in a more streamlined specialist health service. She said:

*In a way they have time for each patient. You are not told that you have ten consultations, and if you haven’t recovered within these ten consultations, we can’t help you.*

#### More fitted

Several interviewees had a long history of illness and experiences with a support system that had provided effective help to varying degrees. Their GP had previously referred them to further services and treatment outside the GP’s surgery. This had not created the more personally fitted service they now experienced with PHT follow-up. The interviewees appreciated the nurses' approach, which included clear and concrete advice. The nurses' broad professional competence and experience-based knowledge of other local healthcare and support services were highlighted as valuable assets. One interviewee with a complex health/life situation who had previously been referred to a psychologist explained how the nurse was a better fit for her. She said:

*[I]f you talk to a psychologist they may not quite understand what somatic illness is all about, but the nurse does […] she understands both my physical and mental condition [ …] and then she knows the local health services that we use, so it is not so difficult for me to explain everything […] she understands what I say and comes with clear advice […] is better at seeing the whole of issues, does not think completely clinical, but sees the whole package.*

#### More coordinated

The interviewees shared various examples of how they experienced a more coordinated care than before. This was attributed to more treatment being given in the GP surgery by the GP and nurse. Several interviewees said they experienced a good collaboration between GP and nurse, which made them feel cared for. Several of the elderly interviewees said the nurse had been of great help and acted as a kind of advocate for them to ensure them better dialogue with, and follow-up from, the home care service. The interviewees also gave examples where other support agencies (such as other therapists and caseworkers at the social security office) had been invited to meet with the patient at the GP’s surgery as part of a coordinated treatment programme where the nurse was often present, or in which the nurse or GP joined the interviewee in meetings with other support agencies outside the GP surgery to back them up, and to ensure access and that they were heard. One interviewee said that it had been great help not being referred for further follow-up with other helpers elsewhere. He said:

*The best thing that has somehow helped me is not having to go back and forth to new people all the time.*

#### More learning

Several interviewee stories illustrated how PHT follow-up is tailored to their individual needs. Patients get help to define their needs and what their treatment plan should be like. Several interviewees pointed out that participating in this co-creation had been a learning process. An interviewee who, with PHT follow-up, had radically changed his lifestyle and lost over 60 pounds of weight said that this would not have been possible without this learning process. Several years ago, his GP had given him a dietary booklet to help him lose weight. This measure was ineffective and did not contribute to any change because he was not able to convert the booklet into a lifestyle change on his own. He said:

*They can make people take a little more responsibility themselves, but they have to learn it. Telling a 70-year-old man that he has to take responsibility for his life – he doesn’t understand what you mean.*

The survey results further indicate that the respondents have an enhanced understanding of, and better master, their health problems since their GP surgery started with a PHT. A majority (74.2 per cent) of the respondents indicated that their health is better followed up than before (see Table 5). Furthermore, more than half of the respondents reported that they understand their health problems better to a large or very large extent, master their health-related issues to a larger extent, feel safe that their health is taken care of, and are more motivated to take care of their own health.

**Table 5:** In comparison to the period before your GP surgery started with primary healthcare teams, to what extent do you find that the follow-up you receive from the GP and nurse contributes to the following (percentage distribution)?

	**Not at all**	**To a little extent**	**To some extent**	**To a large extent**	**To a very large extent**	**Not relevant/ do not know**	**n**
…all in all, your health is better followed up?	0.7	0.7	13.6	38.7	35.5	10.8	279
…your healthcare is better coordinated between different healthcare providers (the GP, home care, the hospital, the Labour and Welfare Organization)?	0.4	4.0	9.4	24.3	22.1	39.9	276
…you understand your health problems better?	0.4	4.3	17.4	39.9	22.8	15.2	276
…you master your health-related issues to a larger extent?	0.7	4.7	21.5	33.6	23.4	16.1	274
…you feel safe that your health is being taken care of to a larger extent?	0.7	0.4	12.8	35.8	40.1	10.2	274
… you master everyday activities to a larger extent?	2.9	6.9	23.4	25.2	13.9	27.7	274
… you have a greater motivation to take care of your own health?	1.5	2.2	19.3	34.9	22.5	19.6	275
…your health is more stable?	3.6	5.8	25.9	28.8	17.9	17.9	274
… all in all, your health is better?	4.0	8.6	26.6	23.0	16.9	20.9	278
… you have an enhanced quality of life?	2.2	8.3	23.6	27.9	18.8	19.2	276

### Information continuity

Among the interviewees, it was predominantly the multi-morbid elderly and interviewees with mental illness who shared experiences of improved information continuity. Several interviewees said that they now found that important concerns they had raised with the nurse were known to the GP and vice versa. They did not have to repeat themselves as they were well used to doing in meetings with different health professionals. This made them feel safe and cared for. One interviewee emphasized that the exchange of information taking place between the GP and the nurse without him being present made the GP in particular seem to understand his situation better than before and this enabled him to give more adapted help than before. He said:

*I notice that they talk about me, even though they have a duty of confidentiality, but they both know very well what is happening to me […] I feel particularly well taken care of […] before, it was like just one or two GP appointments […] now the GP and the nurse have talked together […] then they have understood even more.*

All of the multi-morbid elderly interviewees received home nursing services. They and one next of kin all talked about the nurse having strengthened their position in the dialogue with the home nursing service and to some extent having taken over their position. The next of kin said:

*If there is something about home nursing, we can contact the PHT nurse because she has contact with them. And those of the home nurses who come here know very well who the PHT nurse is. […] I think they have regular meetings.*

However, not all interviewees found that they or the PHT nurse were heard by the home nursing service. One interviewee had repeatedly experienced poor wound treatment from the home care nurses, which did not improve even though the PHT nurse was in close dialogue with the home care nurse about this. However, the patient was uplifted by strategy discussions with the PHT nurse on how they could raise the issue in other ways with the home care nurses and found this of great support in her struggle to get better.

### Relationship continuity

None of the interviewees expressed any dissatisfaction with the fact that their GP had brought a nurse into the relationship between them. Two interviewees with mental illness said that they were initially skeptical about the nurse and had to be persuaded to accept them. For both of them, this skepticism was due to previous bad experiences with healthcare services. One of the interviewees elaborated on this and explained:

*I was probably afraid that [the nurse] would only be someone I had to quarrel with in some way or someone who would not understand, or someone who would make very strict demands or one who in a way could not see my situation.*

Both interviewees had established a good relationship with the nurse. Like other interviewees, they found that the nurse and the GP operated as a functional team.

The respondents also experienced continuity in their relationship with the nurse, and most of them had mainly received follow-up from the same nurse throughout their consultations (87.3 per cent). Table 4 shows that most of the survey respondents found that the GP and the nurse worked as a team in the follow-up on their health (7.9 per cent partly agreed, 71.4 per cent agreed) and that the nurse and GP had a well-functioning collaboration with regard to follow-up on their health (9.2 per cent partly agreed, 74.1 per cent agreed). Further, a majority of survey respondents disagreed or partly disagreed with the statement suggesting that they did not want to be followed up by the nurse at the GP surgery (74.4 per cent disagreed, 4.0 per cent partly disagreed). The respondents differed more in their response when asked about their communication with the GP and the nurse.

Several interviewees reported that, prior to PHT was established, they had experienced good access to, and a short waiting time for, a consultation with their GP. With the PHT, the access had become even better because the nurse was more available for them than the GP. Interviewees said that it was faster to get an appointment with a nurse, they were more accessible on the phone and on digital platforms, and they provided more services at home – especially to the elderly interviewees who, due to impaired health, found it difficult physically to get themselves to the GP surgery. Several interviewees talked about the nurse as a link between them and the GP, and one interviewee said that the nurse strengthened her position in her relationship with the GP. Interviewees said that they could now contact the GP indirectly via the nurse and clarify the need to have direct contact with the GP themselves. One interviewee who had recently developed a chronic illness elaborated on how the PHT contributed to both accessibility and simplification:

*If there's something I need or something I'm wondering about, it's easier to just get an appointment with the nurse, discuss with her and then she takes it to the GP. […] A nurse who is a little more accessible, makes the whole health system a little more accessible and a little easier to deal with.*

The interviewees trusted that it was their GP who controlled what happened to them in the GP surgery. Several among them spoke of the GP as being present even though the patient physically met the nurse and the feeling of having the GP at their back and available if their health condition indicated a need for something more. Several pointed out that it was the GP who had the decision-making authority when it came to their medication. One of them said:

[The GP] is in a way the boss in this scheme.

The survey results also indicate that respondents are satisfied with the availability of both their GP (18.6 per cent satisfied and 67.0 per cent very satisfied) and the nurse (9.5 per cent satisfied and 73.0 per cent very satisfied) as part of the PHT.

## Discussion

The integrated study primarily confirmed a fit between the findings from both types of data. The findings suggest that, seen from a patient perspective, PHTs can strengthen all three types of continuity. Some of our findings in the qualitative study are in line with findings from the few previous qualitative studies conducted to explore patients’ experiences with PHTs. However, none of them have specifically studied aspects of continuity related to PHTs. The interviewees emphasized changes in management continuity and gave various concrete examples of improvements. One notable aspect emphasized by the interviewees was the increased amount of time patients had with the nurse. This allowed for more time to present and discuss their health issues, to participate in treatment decisions, and to carry out treatment. This is in line, to some extent, with Pullon et al. [10], who found that patients receiving care from PHTs appreciated the greater amount of time and attention a PHT nurse could provide. This indicates that PHTs in both a Norwegian and New Zealand setting are about an extended service, where what nurses do mainly come as a supplement to what the GPs do, rather than as a substitute. In this context, increased service quality and more satisfied patients would be expected.

As part of the PHT pilot, two changes have taken place at the GP surgery: the organizational change with the introduction of a PHT with a nurse and the actual supply of resources in the form of nurses. When the patients assess what has improved, their comparison will be based on both changes. Alternatively, the extra resource could be more GP time, in which case the patients would probably also say that they get more time at the GP surgery now than before.

Results from the survey indicate that patients who receive healthcare from PHTs are satisfied with this type of monitoring and wish to receive this type of follow-up. Some respondents highlighted conversations conducted with, and information provided by, the nurse as particularly important for them. Further, the survey indicates that, overall, in comparison with the period before PHTs, most of the respondents felt safer that their health was being taken care of and that their health was being better followed up. However, respondents did not consistently express a strong preference for follow-up from a nurse. For instance, when asked if they found it easier to talk to the nurse than their GP, around a third of the respondents disagreed or partly disagreed with the suggestion that they did, a third neither agreed nor disagreed, and a third agreed or partly agreed.

Seen from a GP’s perspective, this team-based care could be conceived as impairment of their relationship with the patient because personal contact is lost; but seen from the patient’s perspective, our interpretation of the study results is that they find that the nurse helps strengthen their relationship with the GP. There is nothing in the patient interviews indicating that the patients see the PHT as a replacement for the relationship with their regular GP. This relationship seems to be a prerequisite for the PHT to work. Apparently, the patients seem to establish similar one-to-one relationships with the nurse. An interesting aspect is how strong and lasting this relationship will be and in what way it differs from, contributes to, or undermines the relationship between the patient and the GP. At the time of the interviews, the PHTs had not functioned long enough to fully explore aspects related to the continuity of the relationship between patient and nurse over various disease courses.

Szafran et al. [11] found that having other health professionals involved in patient care increased education and knowledge about conditions and how to manage them and through this enhanced patients’ perception of the quality of life. Our findings are in line with this evidence, as the introduction of PHTs has provided a new resource that can initiate learning processes that enable patients to take part in treatment planning and eventually become more able to manage their chronic health condition.

In line with Morgan et al. [13] and Szafran et al. [11], we found that patients felt their access to primary care improved with a PHT in terms of greater ease of contact, enhanced scheduling of appointments, and a decrease in appointment wait times. Patients largely hold the responsibility for initiating their encounter in general practice. Studies from both the UK and Denmark indicate that patients experience an added care barrier and moral pressure not to waste the GP's time, as time is considered a limited resource [12, 34–37]. The interviewees in our study had similar experiences concerning the GP’s limited time resource, but we did not find any indication based on the interviews of experienced care barriers being attributed to regular GP lack of time. However, the changes experienced by the interviewees in treatment and care associated with the introduction of a PHT indicate that a care barrier might have been present as the interviewees had undoubtedly received improved follow-up with the PHT.

There has been some debate regarding the need for PHTs in Norway. Some GPs argue that introducing nurses to GP surgeries to work in teams with GPs is not the way forward. The solution should rather be to increase the number of GPs and reduce the length of patient lists, and in that way be able to give the listed individuals more time with the GP. However, our study suggests that from the patients' perspective, it is seen as an advantage that nurses have entered the GP surgeries with a different competence and a different professional approach to the GP. Several of the interviewees indicated that they are not wondering about questions they would ask the GP or think would be of interest to the GP. The help that they experienced as effective with PHTs had been given by the nurse or in close collaboration between the nurse and the GP. However, as previously mentioned, patients in the survey seemed to vary in their opinion about whether they find it easier to talk to a nurse or their GP.

Relational continuity is highly valued by many patient groups [9], though not universally preferred by all patients [12]. The balance of evidence suggests that continuity leads to more satisfied patients, reduced costs, and better health outcomes [16, 17, 38]. However, relational continuity may also lessen the GP's objectivity, adversely affect decisions on investigation, and generate reluctance to avoid confrontation. Pater-/maternalism can develop, with a loss of autonomy, especially in vulnerable patients, and a patient may become “stuck” in a counterproductive relationship with the GP [39].

Pullon et al. [10] found that PHTs made it possible to avoid vulnerabilities that might arise where only one professional is knowledgeable about a patient’s complex medical history. None of the interviewees in our study talked about this vulnerability. However, several interviewees said that they found it easier to “talk” to the GP via the nurse and that the nurse helped strengthen the relationship with the GP, which may indicate that PHTs can even out the asymmetry known to exist between GP and patient. Berkowitz et al. [12] found diabetes patients to be very open to team-based care, although they emphasized the importance of a single point of contact. The interviewees and the respondents in our studies, with their various chronic and complex conditions, also seem to be open to team-based care at the GP surgery. An important prerequisite for this, however, seems to be that they maintain the one-to-one relationship with their regular GP. Some of the younger interviewees also appreciated that the GP surgery was to a greater extent than before their treatment base.

The Norwegian authorities' piloting of PHTs is part of an international development. PHTs are embedded in larger contextual frameworks where both design and outcome depend on national societal structures in general, and on the structures in the health service in particular. The description of international experiments with PHTs and the evaluations of these show a diversity of organizational models and results [40–44]. The heterogeneity in both health systems and results from international evaluations justify piloting and associated evaluation in the individual countries before whole system changes are eventually initiated. Starfield [45] highlights continuity as one of four basic pillars in a well-functioning primary healthcare service. Her work has served as one of the important inspirations for the Norwegian health authorities. The overriding aim with the introduction of the patient list system in general practice in Norway in 2001 was to promote continuity and to ensure all Norwegians a regular GP. It is therefore not surprising that the interviewees in our study clearly emphasize that the GP's presence in forms a foundation for their PHT support. However, seen in an international context, the Norwegian starting point with such strong relational continuity between GP and patient might seem to be an anachronism. Norwegian research findings must be interpreted within this framework and may to some extents have limited transfer value to other countries.

The interviewees positively emphasize the nurses' broad professional competence, as well as their experience with, knowledge of, and ability to coordinate with the local health service. Several of the PHT nurses have additional education, for example in psychiatry and follow-up of chronic diseases like diabetes and COPD. Maier [9] claims that adequately trained nurses, provide care that leads to higher patient satisfaction and lower hospital (re-) admission when compared to physician-provided care. It is not certain that it will be possible to staff PHTs with this level of nursing competence if PHTs are scaled up and implemented more widely in Norwegian general practice. Furthermore, a main finding in our study is the patients' emphasis on PHTs providing more time in a service that is characterized by overworked GPs with very long working days [46]. This has probably to do with the availability of more resources than previously. There is reason to question whether the seemingly generous time supply would be sustainable if the service were scaled up and spread.

In the years to come, it will become more and more important to facilitate nurses working in the areas of the healthcare system where they are most needed. To ensure this, we believe it is important to have a funding system that supports flexible and context-specific use of nurses. In some municipalities, it may be best to have the nurses working in the GP surgeries, while in others, an alternative organization and use of nurses may be more suitable.

### Strengths and limitations

This study provides an important supplement to the relatively meagre portfolio of research on patients' experiences of transitions to PHTs in general practice. It makes a substantial contribution to the understanding of how patients find that different types of continuity are affected by the fact that their primary health service has transitioned to more team-based care. The study was conducted in a setting where it is possible to study patients' experience of changes in continuity in the transition from single-profession GP practice to PHT follow-up and explore how patients experience this transition, theoretically placed in a continuity framework. The study includes patients followed up by PHTs of different ages and with different diagnoses and ailments. This provides a broad intake that makes it possible to explore experiences across ages and diagnoses/ailments. However, the patient samples both in the qualitative and quantitative studies are small, and it is conceivable that a concentration around a more specific patient group would yield other findings.

The interviewees and the survey participants for our studies were selected by the PHTs. This may have introduced a selection bias in such a way that those interviewed, and the survey participants, are among those most satisfied with PHTs. The more critical voices may not have been heard. Moreover, the chosen survey distribution method leaves room for possible differences in the understanding of which patients were eligible to answer, and we cannot know if all eligible patients were invited to answer.

This must be considered in the interpretation and use of the results. Privacy considerations limited the researchers’ control of the selection process. PHTs are aimed at vulnerable patient groups, and we considered it appropriate to adopt an approach where we contacted patients via health professionals that were known to them and could assess their suitability for inclusion in the studies. Moreover, the survey was distributed by a nurse or a medical assistant at the GP surgeries to ensure that only eligible patients participated. The employees at the GP surgeries may have had a different understanding of who were eligible, and moreover, the GP surgeries may have selectively distributed the survey to patients whom they assumed were pleased with the healthcare they received.

The results are based on qualitative (interviews) and quantitative (survey) data. The mixed-methods design was recommended by Morgan et al. [13] and allows us to ensure that the qualitative findings are supported by quantitative results, and vice versa. The study sheds light on the patients’ opinions in different phases of the pilot, as the qualitative and quantitative information was collected sequentially. This further allowed us to better design the survey to retrieve more relevant information. We interpret it as an unconditional strength that the findings from the qualitative study were predominantly confirmed by the quantitative study results. None of the authors are healthcare professionals. We also do not have experience with extensive health problems. This means that we analyze PHT and associated patient experiences from an outside perspective. We interpret the patients’ statements and are of course not fully capable of capturing the patients’ perspective. This probably limits what was understood in the relevant context but gave us on the other hand an open mind.

## Conclusion

This mixed-methods study indicates that continuity, seen from a patients’ perspective, can strengthen when their primary healthcare encounter with general practice transitions from single-profession GP care to interprofessional team-based care. Findings from patient interviews and survey results reveal that team-based care is perceived as more fitting and better coordinated, offering increased time and more learning, and can bring increased understanding and mastering of patients’ health problems than single-profession GP care. Improvements in information and relational continuity are also experienced among patients. In this study, patients do not see the interprofessional team as a replacement for the one-to-one relationship with their regular GP. The continuation of the patient-GP relationship and the GP’s clear presence in the team seems to be a prerequisite for patients’ positive appreciation of team-based care.

### Supplementary Information

**Additional file 1.**

## Data Availability

The data analysed during the current study are available from the corresponding author on reasonable request.
