# Supplementary material for: Expanding general practice with interprofessional teams: a mixed-methods patient perspective study
Source: BMC Health Serv Res. 2023 Nov 30;23:1327. doi: 10.1186/s12913-023-10322-z (PMC10691031; doi:10.1186/s12913-023-10322-z)
Supplement: Supplementary file 1 — Additional file 1. [file 12913_2023_10322_MOESM1_ESM.pdf]

Interview guide (Qualitative study)

We want to facilitate an open and undirected conversation with patients who receive services from the primary healthcare team (PHT). Initially, we want to make sure that the patient understands who we are specifically talking about when we ask about the PHT (particularly nurses who are new) at the GP surgery they belong to.

Topics that we will get into during the interview:

• The experience of getting follow-up from the PHT for health problems

• Any changes in the follow-up from the PHT compared to regular GP follow-up

• Perceived effects of the follow-up from the PHT team compared with regular GP follow-up

• About the access the GP, the PHT, and the GP surgery

Survey among patients about experiences with follow-up from primary health care teams (Quantitative study)

You are invited to participate in this survey because you are affiliated to a general practitioner's surgery that offers services through primary healthcare teams, and because you have received such services. Primary healthcare teams consist of general practitioners, nurses, and health secretaries, working together to enhance primary care services for patients with large and complex needs.

Consent

Do you consent to participate in this survey?

- ☐ Yes
- ☐ No

Contact with and follow-up from the general practitioner surgery

1. **How many times have you been in contact with your general practitioner or the general practitioner's surgery over the past 12 months?**
   Count all forms of contact: both visit to the general practitioner's surgery, home visits, contact with the general practitioner's surgery over Helsenorge/ the general practitioners web page, sending a letter/ e-mail / message to or contact over the telephone with the general practitioner or nurse (digital consultation).

   - ☐ 0 times
   - ☐ 1 time
   - ☐ 2-5 times
   - ☐ 6-12 times
   - ☐ 13 or more times
   - ☐ *Do not know*

2. **In what/ which circumstance(s) have you been in contact with the general practitioner's surgery over the previous 12 months?** *Select all that apply*

- ☐ Acute disease
- ☐ Medical examination (new illness)
- ☐ Control (previously diagnosed, follow ups, in relation to chronic disease)
- ☐ Certificate of health
- ☐ Vaccine
- ☐ Renewal of prescription
- ☐ Pregnancy
- ☐ General practitioner/ nurse has been om a home visit
- ☐ Other circumstance
- ☐ *Do not know*

3. **Have you been followed up by a nurse at your general practitioner's surgery?**

Select all that apply

- ☐ Yes, as a part of a general practitioner consultation, alongside the general practitioner
- ☐ Yes, in a separate nurse consultation, where I also met the general practitioner
- ☐ Yes, in a separate nurse consultation on the same day as a general practitioner consultation
- ☐ Yes, in a separate nurse consultation on a different day than the general practitioner consultation
- ☐ Yes, the nurse has been on a home visit
- ☐ No → **proceed to question 12**

4. **How many times have you received follow-up from a nurse in the last year during a consultation where the general practitioner has also been (partially) present?**

- ☐ 0 times
- ☐ 1 time
- ☐ 2-5 times
- ☐ 6-12 times
- ☐ 13 times or more
- ☐ *Not applicable/ do not know*

5. **How many times have you received follow-up from a nurse in the last year, as a separate nurse consultation, without the general practitioner being present?**

- ☐ 0 times
- ☐ 1 time
- ☐ 2-5 times
- ☐ 6-12 times
- ☐ 13 times or more
- ☐ *Not applicable/ do not know*

6. **Have you mostly been followed up by the same nurse every time?**

- ☐ Yes
- ☐ No, it depends on the reason for the consultation
- ☐ No, it seems to be random
- ☐ *Not applicable/ Do not know*

7. **How did you experience receiving follow-up from a nurse in a separate nurse consultation?**

- ☐ Very negative
- ☐ Negative
- ☐ Neither negative nor positive
- ☐ Positive
- ☐ Very positive
- ☐ *Not applicable/ Do not know*

8. **What kind of follow-up have you received from the nurse at the general practitioner's surgery?**
Select all that apply

- ☐ Information and training related to how you best can live with your heath issue
- ☐ Conversations about your well-being
- ☐ Medication review
- ☐ Annual check-up (e.g., related to diabetes)
- ☐ Blood test
- ☐ Vaccine
- ☐ Other

Satisfaction with the follow-up from the nurse

9. **In your experience, does the nurse involve you as much as you want in decisions concerning you?**

- ☐ Not at all
- ☐ To a little extent
- ☐ To some extent
- ☐ To a large extent
- ☐ To a very large extent
- ☐ *Not applicable/ do not know*

10. **Does the nurse provide you with sufficient information about your health issues and their treatment?**

- ☐ Not at all
- ☐ To a little extent
- ☐ To some extent
- ☐ To a large extent
- ☐ To a very large extent
- ☐ *Not applicable/ do not know*

11. **Do you feel that the nurse has enough time for you?**

- ☐ Not at all
- ☐ To a little extent
- ☐ To some extent
- ☐ To a large extent
- ☐ To a very large extent
- ☐ *Not applicable/ do not know*

Satisfaction with the follow-up from the general practitioner

12. Do you feel that the general practitioner involves you as much as you want in decisions that concern you?

- ☐ Not at all
- ☐ To a little extent
- ☐ To some extent
- ☐ To a large extent
- ☐ To a very large extent
- ☐ *Not applicable/ do not know*

13. Does the general practitioner provide you with sufficient information regarding your health-related issues and the treatment of these?

- ☐ Not at all
- ☐ To a little extent
- ☐ To some extent
- ☐ To a large extent
- ☐ To a very large extent
- ☐ *Not applicable/ do not know*

14. Do you feel that the general practitioner has enough time for you?

- ☐ Not at all
- ☐ To a little extent
- ☐ To some extent
- ☐ To a large extent
- ☐ To a very large extent
- ☐ *Not applicable/ do not know*

Satisfaction with the general practitioner's surgery

15. Do you perceive the surgery as well-organized?

- ☐ Not at all
- ☐ To a little extent
- ☐ To some extent
- ☐ To a large extent
- ☐ To a very large extent
- ☐ *Not applicable/ do not know*

16. Do you find the other staff at the surgery (besides the general practitioners) to be welcoming and competent?

- ☐ Not at all
- ☐ To a little extent
- ☐ To some extent
- ☐ To a large extent
- ☐ To a very large extent
- ☐ *Not applicable/ do not know*

17. Is it difficult to reach the surgery by phone?

- ☐ Not at all
- ☐ To a little extent
- ☐ To some extent
- ☐ To a large extent
- ☐ To a very large extent
- ☐ *Not applicable/ do not know*

18. Are you greeted with politeness and respect at the surgery reception?

- ☐ Not at all
- ☐ To a little extent
- ☐ To some extent
- ☐ To a large extent
- ☐ To a very large extent
- ☐ *Not applicable/ do not know*

19. All in all, how pleased are you with the availability of the general practitioner?

- ☐ Very dissatisfied
- ☐ Somewhat dissatisfied
- ☐ Neither satisfied nor dissatisfied
- ☐ Somewhat satisfied
- ☐ Very satisfied
- ☐ *Not applicable/ do not know*

20. All in all, how satisfied are you with the availability of the nurse in the primary healthcare team?

- ☐ Very dissatisfied
- ☐ Somewhat dissatisfied
- ☐ Neither satisfied nor dissatisfied
- ☐ Somewhat satisfied
- ☐ Very satisfied
- ☐ *Not applicable/ do not know*

21. All in all, how pleased are you with the overall follow-up from the general practitioner's surgery?

- ☐ Very dissatisfied
- ☐ Somewhat dissatisfied
- ☐ Neither satisfied nor dissatisfied
- ☐ Somewhat satisfied
- ☐ Very satisfied
- ☐ Not applicable/ do not know

22. Consider the following statements

Place one mark on each line. If the description does not apply to you, check "Not applicable/Do not know".

	Strongly disagree	Partly disagree	Neither agree nor disagree	Partly agree	Strongly agree	Not applicable / do not know
**The collaboration between the general practitioner and nurse in the follow-up of my health works efficiently**						
**The general practitioner and nurse at the *general practitioner surgery* work together as a team in my follow-up**						
**The general practitioner, nurse, and health secretary at the *general practitioner surgery* work together as a team in my follow-up**						

23. Compared to the period before your general practitioner's surgery introduced the primary healthcare team, to what extent do you feel that the follow-up from the general practitioner and nurse at the surgery contributes to that...

Place one mark on each line. If the description does not apply to you, check "Not applicable/Do not know".

	Not at all	To a little extent	To some extent	To a large extent	To a very large extent	Not applicable /do not know
…you overall receive better health follow-up?						
…the follow-up of your health is better coordinated among different entities (e.g., general practitioner, home care, hospital, NAV)?						
…you better understand your health issues?						
…you better manage your health issues?						
…you feel a greater sense of security that you are being well taken care of?						
… you are more able to engage in daily activities?						

… you have gained greater motivation to take care of your own health?						
…you have a more stable health condition?						
… you have experienced overall improvement in health?						
… you have achieved a better quality of life?						

Preferences for healthcare provided by the general practitioner’s surgery

Typically, the patient contacts the general practitioner's surgery when follow-up is needed. Another option is that the general practitioner or nurse initiates contact if they believe the patient requires follow-up (i.e., without the patient initiating contact first).

24. Have you experienced being contacted by the general practitioner's surgery without you initiating contact first, in relation to...? *Select all that apply*

- ☐ That you have a specific diagnosis (e.g., for an annual check-up related to the diagnosis)
- ☐ A severe illness you have had
- ☐ You are in a specific age group
- ☐ Vaccination
- ☐ Lifestyle change
- ☐ It has been a long time since you contacted the general practitioner’s surgery yourself
- ☐ You have been discharged form the hospital or other healthcare institution
- ☐ Other follow-up
- ☐ I have been contacted in this way, but I am not aware of the reason
- ☐ I have not experienced to be contacted in this way → **proceed to question 26**
- ☐ *Do not know*

25. How did you experience being contacted this way?

- ☐ Very negative
- ☐ Negative
- ☐ Neither negative nor positive
- ☐ Positive
- ☐ Very positive
- ☐ Not applicable/ Do not know

26. How do you think you would experience being contacted by the general practitioner's surgery without you initiating contact first?

- ☐ Very negative
- ☐ Negative
- ☐ Neither negative nor positive
- ☐ Positive
- ☐ Very positive
- ☐ Not applicable/ Do not know

27. Consider the following statements

Place one mark on each line. If the description does not apply to you, check "Not applicable/Do not know".

	Strongly disagree	Partly disagree	Neither agree nor disagree	Partly agree	Strongly agree	Not applicable / do not know
**I do not wish for follow-up from a nurse at the general practitioner's surgery**						
**The nurse has more time for me than the general practitioner**						
**The general practitioner understands my health concerns better than a nurse**						
**It is easier for me to discuss things I am unsure about with the nurse than with the general practitioner**						
**The nurse provides useful advice that I would not receive from the general practitioner**						

28. Do you have any additional comments regarding receiving healthcare from a nurse at the general practitioner's surgery?

……

……

……

……

Background information

29. Which general practitioner's surgery are you affiliated with?

- ☐ Austevoll legesenter
- ☐ Best Helse
- ☐ Brumunddal legesenter
- ☐ Brynklinikken fastlegesenter
- ☐ Brynsenglegene
- ☐ Bystranda legesenter
- ☐ Eid legekontor
- ☐ Gransdalen legesenter
- ☐ Herredshuset legesenter
- ☐ Legegruppa SMS
- ☐ Moelv legesenter
- ☐ Nærbø helsesenter
- ☐ Rana lokalmedisinske senter
- ☐ Sagene lokalmedisinske
- ☐ Sandens medisinske senter
- ☐ Seljord helsesenter
- ☐ Sørlandsparken legesenter
- ☐ Do not wish to answer

30. What is your age?

- ☐ 18–39 years
- ☐ 40–59 years
- ☐ 60–79 years
- ☐ 80 years or older
- ☐ Do not wish to answer

31. What is your gender?

- ☐ Male
- ☐ Female
- ☐ Other
- ☐ Do not wish to answer

32. What is your highest achieved level of education?

- ☐ Elementary school
- ☐ High school
- ☐ College/university (up to 3 years)
- ☐ College/university (4 years or more)
- ☐ Do not wish to answer

33. Do you have any of the following long term medical conditions? *Select all that apply*

- ☐ High blood pressure (hypertension)
- ☐ Heart disease, including heart attack
- ☐ Diabetes
- ☐ Asthma or other chronic lung diseases like chronic bronchitis, emphysema, or COPD
- ☐ Depression, anxiety, or other mental health issues
- ☐ Substance abuse problems
- ☐ Cancer
- ☐ Problems with muscle and/or skeleton, including joints or arthritis
- ☐ Previously had a stroke
- ☐ Other long-term health problems/conditions
- ☐ No long-term health problems/conditions
- ☐ Do not wish to answer

34. All in all, how would you describe your own health?

- ☐ Very bad
- ☐ Pretty bad
- ☐ Neither bad nor good
- ☐ Pretty good
- ☐ Very good
- ☐ Do not wish to answer

35. How long have you had the same general practitioner?

- ☐ Less than a year
- ☐ 1-4 years
- ☐ 5 years or more
- ☐ I do not have a general practitioner at the moment
- ☐ Do not know/ do not wish to answer
